# Effects of Lifetime Exposure to Sports-Related Head Impacts on Brain Injury and Inflammatory Blood Biomarkers Among Former Middle-Aged Athletes

**DOI:** 10.1177/08977151251362101

**Published:** 2025-08-05

**Authors:** Grace O. Recht, Giselle Lima-Cooper, Claire V. Buddenbaum, Sage H. Sweeney, Zachary S. Bellini, Sharlene D. Newman, Dibyadyuti Datta, Keisuke Kawata

**Affiliations:** ^1^ Department of Kinesiology, Indiana University School of Public Health-Bloomington, Bloomington, Indiana, USA.; ^2^ Department of Pediatrics, Indiana University School of Medicine, Indianapolis, Indiana, USA.; ^3^ Alabama Life Research Institute, University of Alabama, Tuscaloosa, Alabama, USA.; ^4^ Program in Neuroscience, The College of Arts and Sciences, Indiana University, Bloomington, Indiana, USA.

**Keywords:** blood biomarker, chronic traumatic encephalopathy, concussion, subconcussive head impacts, tau, traumatic brain injury

## Abstract

Repetitive head impacts from contact sports are associated with an increased risk of neurodegenerative conditions. While studies have examined acute and chronic outcomes in young and deceased athletes, research on middle-aged former athletes remains limited. We employed multiplex biomarker approaches to examine whether brain injury and systemic inflammatory blood biomarkers are reflective of ≥10 years of participation in contact sports in retired, middle-aged amateur athletes. This cross-sectional study included a cohort of 41 former contact athletes (32 male, 9 female) and 22 age- and sex-matched noncontact athletes (14 male, 8 female). Blood biomarkers of brain injury, including glial fibrillary acidic protein, ubiquitin C-terminal hydrolase L1 (UCH-L1), tau, and neurofilament light (NfL), alongside 18 systemic inflammatory markers, were examined via linear regression models with age and concussion history as covariates. Our analyses revealed no significant differences in brain injury blood biomarkers between groups. However, increasing age was associated with increased NfL levels in contact athletes, while greater concussion history correlated with elevated UCH-L1 and tau in contact athletes only. Contact athletes exhibited significantly increased levels of systemic inflammatory markers, including IL-8, CCL-2, CCL-3, IL-2, VCAM-1, and S100B. While brain injury blood biomarkers did not differ between groups, the association between age, concussion history, and increased NfL, UCH-L1, and tau levels in the contact group suggests potential long-term neural consequences of repetitive head impacts. Elevated systemic inflammatory markers potentially highlight a chronic inflammatory response in former contact athletes, underscoring the need for continued monitoring and interventions to mitigate neurodegenerative risk.

## Introduction

Years of exposure to repetitive head impacts (RHI) from contact sports significantly increase the risk of early onset of neurodegenerative conditions such as chronic traumatic encephalopathy (CTE)^1^ and Alzheimer’s disease (AD),^2^ often mediated by neuroinflammatory pathways.^3–6^ Over the past decade, researchers have explored the mechanisms, neurobiology, and clinical consequences of sport-related RHI through prospective studies in adolescents and young adults.^7,8^ In contrast, CTE researchers have employed retrospective analyses to investigate the biobehavioral and neuropathological correlates of RHI-induced neurodegenerative conditions in deceased individuals.^9^ These complementary approaches provide critical insights into both the acute and long-term consequences of RHI. However, the lack of studies focusing on middle-aged adults leaves a critical gap in understanding who may be predisposed to RHI-induced neurodegenerative conditions. Identifying at-risk individuals within this middle-aged adult population remains a key challenge for advancing early detection and intervention strategies.

The quest to identify objective surrogates of brain health has led to the discovery of blood-based biomarkers that offer a minimally invasive approach while reflecting cellular, structural, and metabolic changes. A panel of brain injury blood biomarkers, including glial fibrillary acidic protein (GFAP), ubiquitin C-terminal hydrolase L1 (UCH-LI), tau protein, and neurofilament light (NfL), represents the forefront of brain injury diagnostics. GFAP, an intermediate filament, maintains astrocyte structural integrity, while UCH-L1 is a cellular enzyme essential for neuronal homeostasis.^10,11^ Together, GFAP and UCH-L1 have demonstrated strong diagnostic utility in differentiating individuals with concussions or mild traumatic brain injury (TBI) from healthy controls with 100% sensitivity and 67% specificity^12–14^ and in identifying patients with intracranial bleeding.^15^ Tau and NfL are key structural proteins within neuronal axons, contributing to microtubules and neurofilaments. Elevated plasma tau levels have been observed across TBI severities,^16,17^ with the magnitude of acute tau elevation correlating with prolonged recovery duration in individuals with concussion.^18^ Conversely, NfL exhibits high sensitivity in detecting both acute RHI^7,19^ and chronic neurological sequelae of TBI or RHI. A 5-year longitudinal study of a TBI cohort (55% mild, 33% moderate, 15% severe) found that serum NfL levels at 30 days post-injury had a diagnostic accuracy of 84–92%. At 5 years post-injury, NfL levels remained significantly elevated compared with healthy individuals, and these persistent elevations correlated with brain atrophy and axonal degeneration, as assessed by diffusion-weighted magnetic resonance imaging techniques.^17^ However, it remains unclear whether these brain injury biomarkers can reliably reflect chronic neurological stress resulting from years of RHI exposure in middle-aged former athletes.

Emerging evidence highlights a complex interaction between brain trauma and systemic dysfunction, wherein chronic inflammation serves as a central mediator for alterations in cardiovascular health, gastrointestinal functions, and endocrine balance.^20,21^ Systemic inflammatory markers, such as interleukins (e.g., IL-1, IL-2, and IL-8) and chemokines (e.g., CCL-2 and CCL-3), have been extensively studied in a variety of contexts such as trauma,^22^ aging,^23^ and chronic diseases.^24^ More specifically, TBI initiates a systemic inflammatory response through a complex cascade of molecular and cellular events. Damaged brain tissue releases damage-associated molecular patterns, such as high mobility group box 1, which activate microglia and astrocytes in the brain parenchyma. This activation further leads to the release of inflammatory cytokines and chemokines, promoting the recruitment of peripheral immune cells and exacerbating systemic inflammation.^25^ The evidence of chronic microglial activation and cytokine release has been observed in individuals with an extensive history of concussive and subconcussive RHI.^26^ Middle-aged, former contact sports athletes with years of exposure to RHI may represent a particularly vulnerable cohort, with heightened risks for inflammation-related neurological conditions, such as CTE or AD.

The current pilot cohort study sought to explore this knowledge gap by investigating the impact of lifetime exposure to sports-related RHI in middle-aged, retired amateur athletes through a response profile of brain injury blood biomarkers, as well as systemic inflammatory markers. We hypothesized that middle-aged adults with 10 or more years of contact sports participation would exhibit elevated levels of brain injury blood biomarkers (GFAP, UCH-L1, tau, and NfL) as compared with their noncontact sports controls. Our exploratory analysis focused on a panel of 18 inflammatory makers to test the hypothesis that the contact group would exhibit significantly elevated levels of cytokines and chemokines compared with noncontact athletes.

## Methods

### Participants

**FIG. 1.**
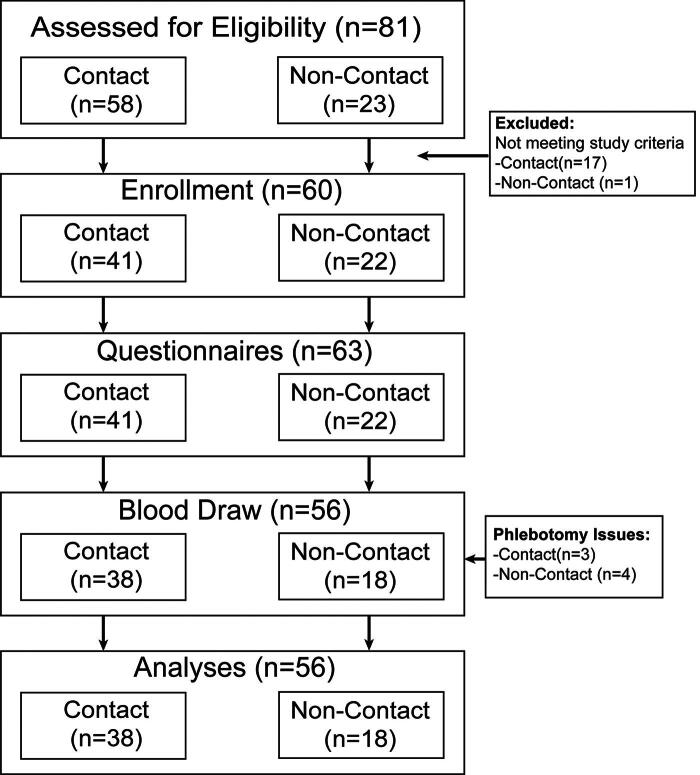
Study flowchart.

This cohort study included 41 contact sport participants (32 male, 9 female) and 22 age- and sex-matched noncontact sport participants (14 male, 8 female). Potential participants were recruited by emails to community partners, social media posts, and iConnect through Indiana CTSI. Data collection occurred between February 2023 to September 2023. Inclusion criteria for the contact group were being male or female between the ages of 30 and 60 and participation in at least 10 years of organized contact sports. A contact sport was operationally defined as sports that have routine body-to-body contact that is expected as part of the sport played, including sports such as football, rugby, and soccer. For the noncontact group, inclusion criteria consisted of being male or female between the ages of 30 and 60, participation in at least 10 years of organized noncontact sports, and no history of contact sport participation. Noncontact sports were operationally defined as sports where body-to-body contact is rare and not expected as part of the game, including sports such as swimming, cross-country/track, and tennis. Exclusion criteria for both groups were any head, neck, or facial injury in the 6 months prior to study participation, pregnancy, history of any neurological disorders, impaired decisional capacity, metal implants in the head, and any implanted electromagnetic devices. Figure 1 describes the flow of the study. The Indiana University’s Institutional Review Board approved the study protocol (no. 17763), and all participants provided informed consent before participating in the study procedures. Once potential participants completed consent meetings, a series of questionnaires were administered to obtain demographic information, athletic history, and concussion history. While concussion history was self-reported, they had to be diagnosed by a medical professional.

### Blood biomarker assessments

On testing day, 4 mL of venous blood was collected into an EDTA vacutainer tube. Plasma was then separated by centrifuge (1500 *g*, 15 min, 4°C) and stored at −80°C until analysis. Brain injury blood biomarkers, including GFAP, UCH-L1, tau, and NfL, were analyzed by the Human Neurology 4-Plex A assay on a Quanterix SR-X system. The mean intraassay coefficients of variation for the samples were 7.8% for GFAP, 2.9% for UCH-L1, 2.2% for tau, and 0.3% for NfL. The lowest detection limit of the assay was 0.771 pg/mL for GFAP, 12.0 pg/mL for UCH-L1, 0.112 pg/mL for tau, and 0.386 pg/mL for NfL.

A Human Magnetic Luminex Assay was designed using the assay service from R&D Systems. The custom Luminex multiplex assay used a 3-fold plasma sample dilution to quantify a panel of inflammatory cytokines (IL-6, IL-8, IL-1α, IL-2), chemokines (CCL-2, CCL-3, CCL-5), neuronal and glial injury markers (BDNF, S100B, NSE-2, α-synuclein), and vascular integrity markers (Ang-1, Ang-2, neuropilin-1, VCAM-1, ICAM-1, thrombomodulin, CD31) in plasma samples. The mean intraassay coefficients of variation for the samples were 3.8% for IL-6, 4.0% for BDNF, 6.4% for IL-8, 7.4% for S100B, 6.5% for CCL-2, 3.7% for angiopoietin-2, 0.3% for neuropilin-1, 2.2% for CCL-3, 0.8% for CCL-5, 6.0% for IL-1α, 7.9% for IL-2, 1.4% for CD31, 0.9% for thrombomodulin, 8.5% for neuron-specific enolase, 1.0% for VCAM-1, 4.0% for ICAM-1, 7.7% for α-synuclein, and 1.9% for angiopoietin-1. The lowest detection limit of the assay was 16 pg/mL for IL-6, 90 pg/mL for BDNF, 27 pg/mL for IL-8, 26 pg/mL for S100B, 9.5 pg/mL for CCL-2, 26 pg/mL for angiopoietin-2, 53 pg/mL for neuropilin-1, 13 pg/mL for CCL-3 18 pg/mL for CCL-5, 11 pg/mL for IL-1α, 33 pg/mL for IL-2, 12 pg/mL for CD31, 59 pg/mL for thrombomodulin, 52 pg/mL for neuron-specific enolase, 19 pg/mL for VCAM-1, 22 pg/mL for ICAM-1, 47 pg/mL for α-synuclein, and 26 pg/mL for angiopoietin-1.

### Statistical analysis

Differences in demographic variables between the contact and noncontact groups were assessed by two-tailed independent-samples *t*-tests for continuous variables (age, number of previous concussions, and years of sport participation) and chi-square analysis for categorical variables (sex, race, and ethnicity). Group differences in brain injury biomarkers (GFAP, UCH-L1, tau, and NfL) were initially inspected by unpaired *t*-tests and then further examined by linear regression models, in which the four biomarkers were set as the primary outcomes. Each model was adjusted for covariates, including age and concussion history. The significance level was set at *p* < 0.0125 to reflect four primary outcomes. For the exploratory analysis using 18 inflammatory markers, we employed similar linear regression models as the primary analysis. The models were adjusted for age and number of concussions as covariates. Given the exploratory nature of these biomarkers, the significance level was set at *p* < 0.05. All primary and exploratory models were summarized by providing a contrast estimate with its 95% confidence interval (CI) and *p* value in the following format: (β estimate [95% CI: low CI, high CI], *p* value). All analyses were conducted using R, version 4.4.2 (R Project for Statistical Computing) using the nlme and emmeans packages.

## Results

### Demographics

A total of 56 participants were included in this analysis (contact *n* = 38, noncontact *n* = 18). Of the 38 contact athletes (age = 42.1 ± 9.3 years), 28 (73.7%) were male. Of the 18 noncontact athletes (age = 43.8 ± 9.0 years), 11 (61.1%) were male. Participants in both groups were predominantly White (92.1–94.4%). The contact group had 15.0 ± 4.9 years of contact sports experience, while the noncontact group had 16.8 ± 6.2 years of noncontact sports experience. The only significant difference in demographics was the number of previous concussions, with the contact group having significantly more concussions than the noncontact group (*p* = 0.05). Demographics are summarized in Table 1.

**Table 1.** Group Demographics

Group	Contact sport	Noncontact sport	*p* Value
*n*	38	18	—
Sex (%)	28M (73.7%)	11M (61.1%)	0.52
Age, years	42.1 (9.3)	43.8 (9.0)	0.51
Race, *n* (%)			0.79
** **White	35 (92.1)	17 (94.4)	—
** **Black/African American	0 (0.0)	0 (0.0)	—
** **Asian	2 (5.3)	1 (5.6)	—
** **Multiracial	1 (2.6)	0 (0.0)	—
Ethnicity, *n* (%)			0.70
** **Not Latino/Hispanic	38 (100.0)	17 (94.4)	—
** **Latino/Hispanic	0 (0.0)	1 (5.6)	—
No. of previous concussions			0.05^a^
** **0, *n* (%)	23 (60.5)	15 (83.3)	—
** **1, *n* (%)	6 (15.8)	1 (5.6)	—
** **2, *n* (%)	2 (5.3)	1 (5.6)	—
** **3+, *n* (%)	7 (18.4)	1 (5.6)	—
Organized sport experience,^a,b^ *n* (%)			0.28
** **Football	20 (52.6)	—	—
** **Soccer	18 (47.4)	—	—
** **Wrestling	8 (21.1)	—	—
** **Hockey	4 (10.5)	—	—
** **Baseball	—	13 (72.2)	—
** **Cross-country/track	—	6 (33.3)	—
** **Volleyball	—	5 (27.8)	—
** **Tennis	—	4 (22.2)	—
Years since retirement, years	18.8 (10.8)	21.9 (6.0)	0.26

^a^ The four most participated in sports.

^b^ Numbers and percentages are equal to more than 100% due to individuals participating in multiple sports.

### Brain injury blood biomarkers

**FIG. 2.**
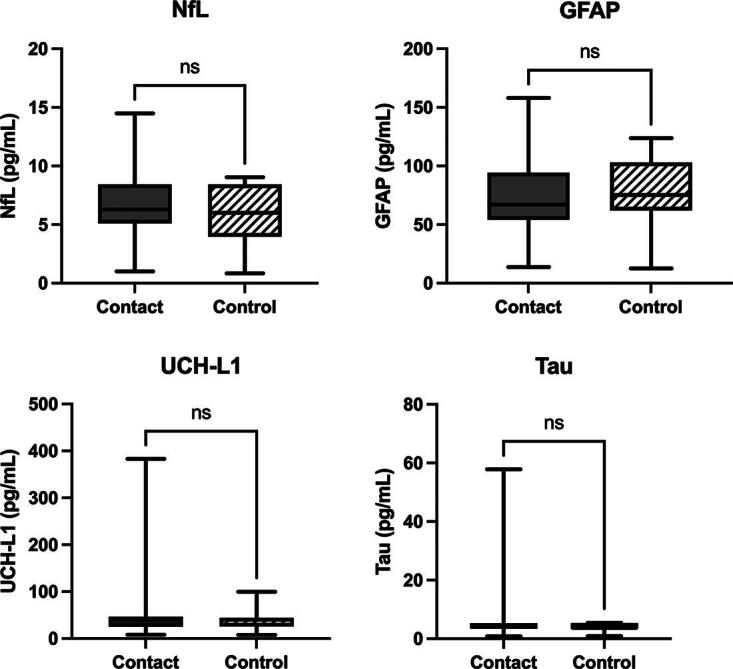
Group differences in brain injury blood biomarkers. There was no significant group difference in any of the brain injury blood biomarkers. GFAP, glial fibrillary acidic protein; NfL, neurofilament light; UCH-L1, ubiquitin C-terminal hydrolase L1.

Contrary to our hypothesis, both groups exhibited similar levels of GFAP, UCH-L1, tau, and NfL (contact vs. noncontact, GFAP: 74.9 ± 33.9 pg/mL versus 82.2 ± 30.4 pg/mL; UCH-L1: 113.5 ± 121.6 pg/mL versus 102.7 ± 151.1 pg/mL; tau: 11.7 ± 12.5 pg/mL versus 8.9 ± 11.6 pg/mL; and NfL 7.1 ± 3.8 pg/mL versus 6.2 ± 2.6 pg/mL; Fig. 2). It is worth noting that post-hoc analyses revealed that increasing age demonstrated significantly increased NfL levels (0.15 pg/mL [0.04, 0.24], *p* = 0.009) in former contact athletes. Whereas additional post-hoc analyses exposed that elevated numbers of previous concussions were associated with significant increases in UCH-L1 and tau (UCH-L1: 26.9 pg/mL [10.8, 41.1], *p* = 0.001, tau: 4.1 pg/mL [2.1, 6.2], *p* = 0.0002) in former contact athletes. Please see Supplementary Table S1 for regression output regarding group differences and Supplementary Table S2 for average biomarker values.

### Systemic inflammatory markers

**FIG. 3.**
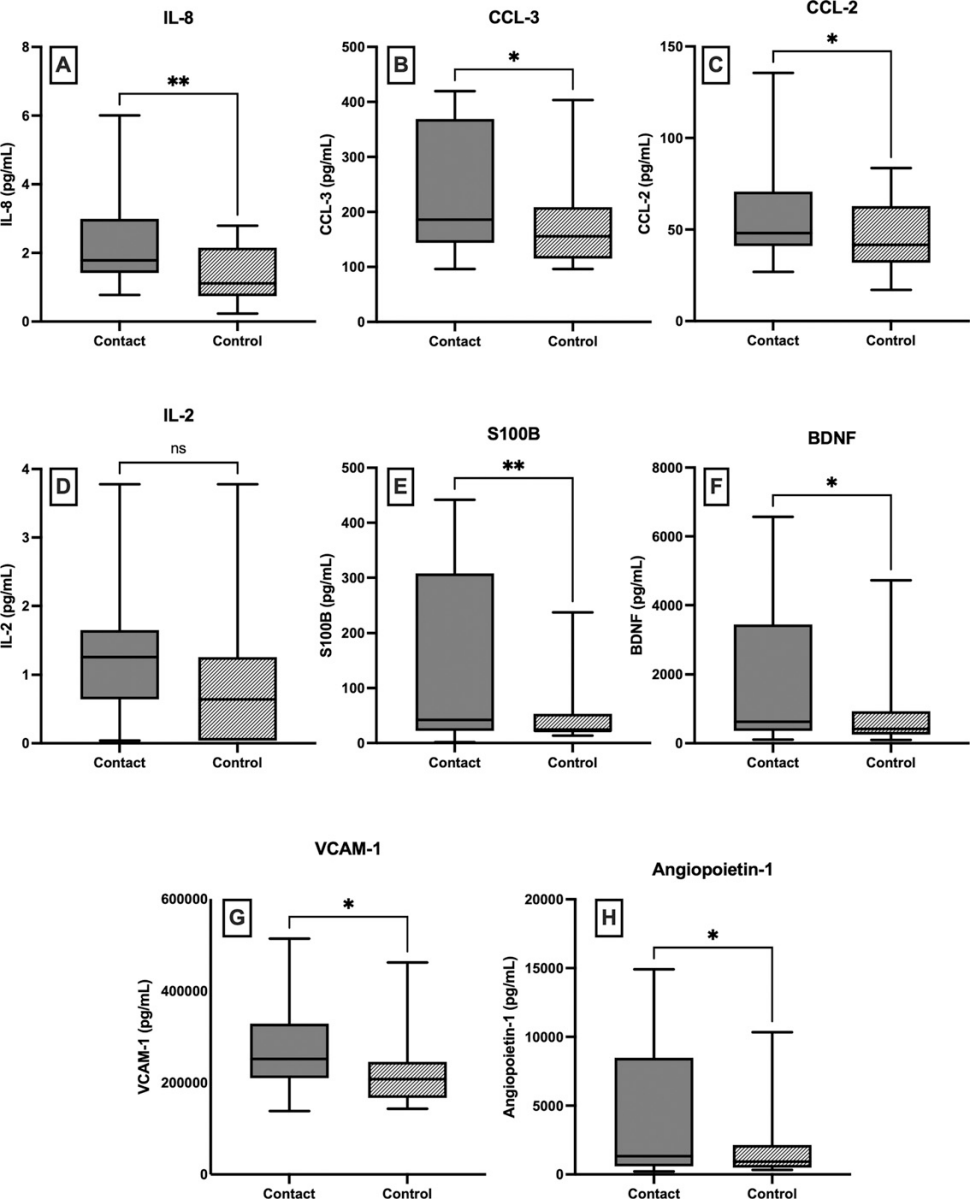
Systemic markers exhibit group differences. Significant group differences were observed in a number of markers, with all markers exhibiting greater levels in the contact group, compared with the noncontact group. Inflammatory cytokines and chemokines include IL-8 **(A)**, CCL-3 **(B)**, CCL-2 **(C)**, IL-2 **(D)**, as well as astrocyte activation marker S100B **(E)** and neurotrophic factor BDNF **(F)**. Both vascular markers reflective of inflammation, VCAM-1 **(G)**, and vascular development marker angiopoietin-1 **(H)** showed elevations in the contact group compared with the noncontact group.

**FIG. 4.**
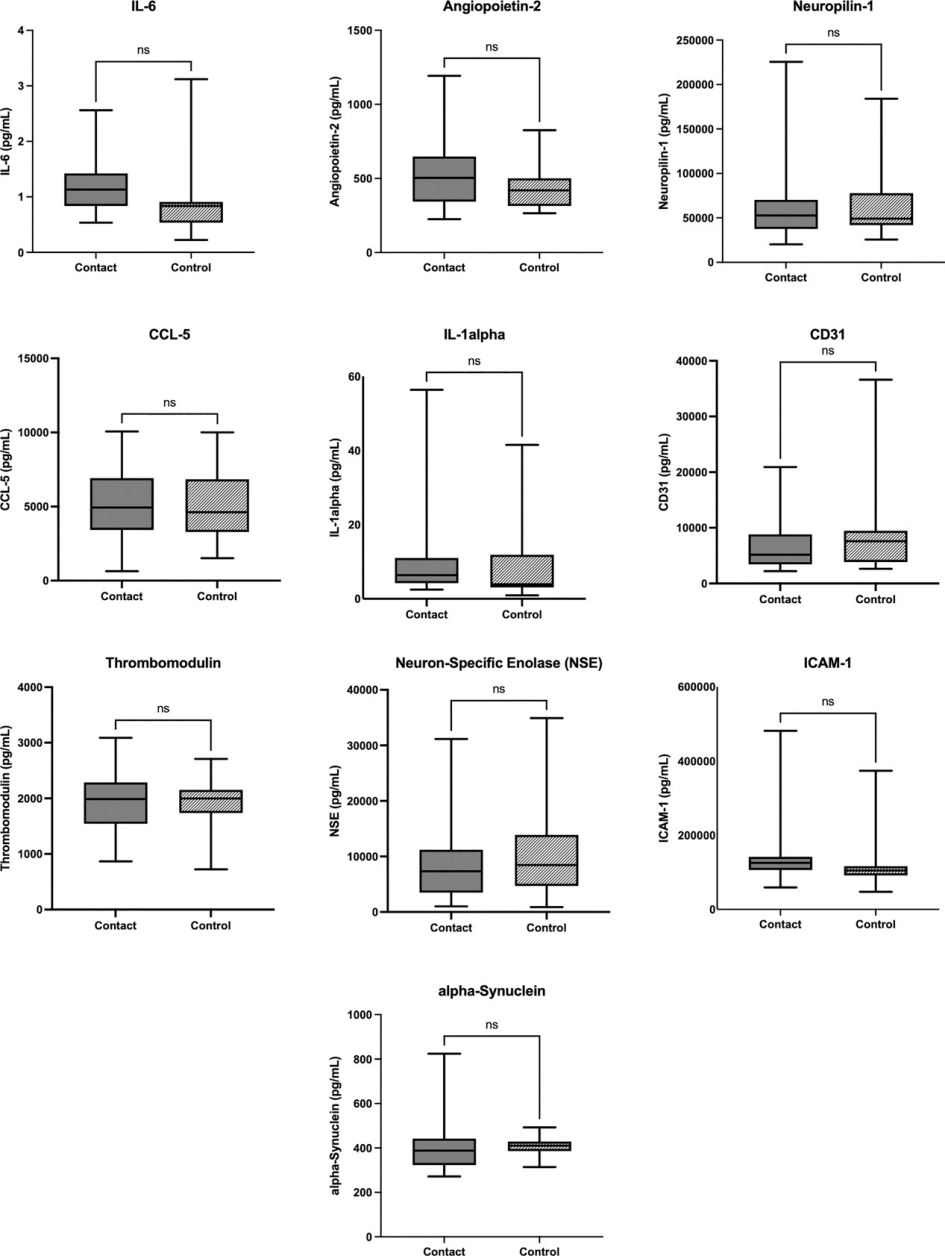
Systemic markers that are not reflective of group differences. Ten markers reflective of inflammatory and vascular and neuronal/synaptic health did not differ between groups.

Mixed results were found in the 18 exploratory markers. Six markers (IL-8, S100B, CCL-2, CCL-3, IL-2, and VCAM-1) demonstrated significantly increased levels in contact athletes as compared with the noncontact athletes (IL-8: 0.9 pg/mL [0.3, 1.5], *p* = 0.007; S100B: 74.3 pg/mL [−1.7, 150.3], *p* = 0.05; CCL-2: 14.1 pg/mL [−0.4, 28.6], *p* = 0.05; CCL-3: 59.5 pg/mL [−2.3, 121.3], *p* = 0.05; IL-2: 0.5 pg/mL [−0.01, 1], *p* = 0.05; VCAM-1: 56,239.3 pg/mL [8756.1, 103,722.6], *p* = 0.02; Fig. 3). The contact group had elevated levels of BDNF (contact 1912.0 ± 2109.1 pg/mL versus noncontact 902.4 ± 1178.2 pg/mL, *p* = 0.02) and angiopoietin-1 (contact 4068.5 ± 4500.6 pg/mL versus noncontact 1889.6 ± 2504.8 pg/mL, *p* = 0.02) compared with the noncontact group. The significant group differences of BDNF and angiopoietin-1 became nonsignificant when age and number of concussions were included as covariates in the model. The rest of the markers showed no significant group differences (IL-6, angiopoietin-1, neuropilin-1, CCL-5, IL-1α, CD31, thrombomodulin, neuron-specific enolase, ICAM-1, and α-synuclein; Fig. 4). Please see Supplementary Table S1 for regression output regarding nonsignificant group differences and Supplementary Table S2 for average biomarker values.

## Discussion

The novelty of this study lays with comprehensive examinations of brain-derived and systemic blood biomarkers in cohorts of middle-aged, retired amateur-level contact and noncontact athletes. There were several key findings. First, as opposed to our hypothesis, there were no significant differences in brain injury blood biomarkers (GFAP, UCH-L1, NfL, and tau) between contact and noncontact athletes. However, former contact athletes with a history of more concussions exhibited increased levels of UCH-L1 and tau. Second, contact athletes had significantly increased levels of systemic biomarkers, including IL-8, CCL-2, CCL-3, IL-2, VCAM-1, and S100B, compared with their noncontact counterparts. The remaining inflammatory markers showed no significant variations between groups. Overall, while contact sports did not lead to clear differences in brain injury biomarkers, they were associated with increased levels of certain inflammatory cytokines and chemokines, suggesting that participation in a decade or more of contact sports may trigger chronic inflammatory responses.

Contrary to our hypothesis, the panel of brain injury blood biomarkers did not capture neural burden from lifetime RHI exposure. Despite GFAP, UCH-L1, NfL, and tau being four of the most well-studied and effective markers in gauging the severity of neural injuries, our findings point to the limitations of these biomarkers in reflecting chronic stages of neuronal health. These markers all reflect different aspects of neural damage, with NfL and tau serving as indicators of axonal injury, GFAP as a measure of astrocytic response, and UCH-L1 as a marker for neuronal damage.^27^ Nevertheless, a critical limitation of these biomarkers lies in their temporal dynamics and sensitivity, which hinder their long-term applicability for assessing chronic neurodegenerative conditions. Studies have demonstrated that each of these biomarkers rises acutely following head impacts, peaking within hours to days post-injury.^27^ For example, GFAP and UCH-L1 exhibit rapid elevations within an hour post-injury, making them highly sensitive in the immediate phase of a TBI.^13^ Similarly, total tau (t-tau) and NfL concentrations increase following structural axonal damage, with NfL potentially persisting for weeks due to its slower clearance from circulation.^17^ Despite these acute-phase sensitivities, longitudinal studies indicate that these biomarkers normalize over time, often within weeks to months post-injury.^28^ For instance, a study that examined biomarker levels in patients with TBI found that while both GFAP and NfL levels remained elevated for 6–12 months following the injury, levels appeared to normalize when tested again 6–13 years post-injury.^29^ Additionally, a longitudinal study on t-tau following TBI found that while levels remained elevated for 7 days post-injury, they normalized within 1 year of the injury.^30^ Due to its short half-life, UCH-L1 may have limited utility for long-term monitoring.^27^ Specifically, a study assessing individuals with TBI of varying severities at acute and chronic timepoints (up to 5 years post-injury) found no significant differences in UCH-L1 levels between patients with TBI and healthy controls.^17^ The absence of sustained biomarker elevation suggests that while these markers are valuable for diagnosing acute neural injuries, they may have limited applicability in monitoring chronic neurodegenerative processes.

Despite no group differences, we found that NfL levels increased with age in former contact sport athletes, whereas this pattern was absent in former noncontact athletes. Previous research has shown that RHI contributes to a progressive rise in NfL levels, reflecting neurodegenerative progression.^31^ Middle-aged amateur athletes with a background in contact sports may be accumulating axonal damage over time, with the effects becoming more pronounced as they age due to repeated concussive and subconcussive impacts that compromise neuronal integrity. Additionally, aging itself is associated with increased neuroinflammation, reduced neuroplasticity,^32^ and elevated NfL levels.^33^ However, the absence of rising NfL in former noncontact athletes may indicate either an interactive effect of aging and RHI on axonal integrity or enhanced cardiovascular fitness through noncontact sports participation may mitigate age-related axonal degeneration.^34^

Similar to the role of age in NfL levels, a history of more concussions was associated with elevated levels of both UCH-L1 and tau in former contact sport athletes, but not in former noncontact athletes. UCH-L1, essential for protein degradation and neuronal repair, is released into the bloodstream following trauma,^25^ and its sustained elevation supports the idea that repeated concussions can contribute to cumulative neuronal stress and increased proteolytic activity, potentially driving chronic alterations in neuronal homeostasis.^14^ More specifically, repeated concussions may cause structural damage and functional impairments, leading to the accumulation of damaged proteins that would require degradation by the ubiquitin–proteosome system.^33^ Similarly, tau, a key microtubule-associated protein, also increased with concussion history in former contact athletes. Recurrent concussions are also known to contribute to disruption of the axonal microtubules and chronic neuroinflammation, further exacerbating tau pathology and increasing the detectability in the periphery.^29^ Elevated tau levels have been observed in professional athletes who have been exposed to RHI, reinforcing the idea that cumulative exposure to both concussive and subconcussive head impacts may lead to long-term neurodegenerative processes.^35^ Interestingly, specific phosphorylated isoforms of tau, including p-tau181, p-tau217, and p-tau231, have been emerging as potential indicators of neurodegenerative processes linked to RHI. These isoforms have shown promise in distinguishing tau pathology in other neurodegenerative conditions such as AD.^36^ Additionally, p-tau181 and p-tau217 have demonstrated sensitivity in detecting early tau abnormalities associated with RHI.^37^ A future study is encouraged to incorporate these tau isoforms to study the potential neurological burden from RHI in middle-aged former athletes.

The current study yielded an important discussion point regarding RHI’s influence on inflammatory cytokines and chemokines, as multiple systemic inflammatory markers were found to be elevated in former contact sport athletes as compared with noncontact sport athletes. Specifically, IL-8, S100B, CCL-3, and VCAM-1 levels were all significantly increased in former contact athletes. These results are consistent with existing literature that has linked exposure to RHI and mechanical stress in contact sports to chronic neuroinflammatory responses.^26,35^ Elevated IL-8, which is a pro-inflammatory cytokine, may reflect a persistent immune activation, which could potentially contribute to neurodegenerative processes.^36,37^ Similarly, an increase in S100B, which is a biomarker of blood–brain barrier (BBB) disruption and astrocyte activation, could suggest heightened neural distress in contact athletes,^38–40^ reinforcing concerns about long-term neurological consequences.^41^ Furthermore, CCL-3, which is a chemokine that is involved in leukocyte recruitment, may indicate immune cell infiltration in response to past injuries.^42^ The significantly elevated VCAM-1 levels could highlight potential endothelial dysfunction and chronic vascular inflammation.^43^ Notably, these findings persisted despite controlling for concussion history, suggesting that repeated subconcussive RHI, rather than diagnosed concussions alone, may drive chronic inflammation in the former contact athletes.

Additional systemic inflammatory biomarkers, specifically CCL-2, IL-2, and thrombomodulin, were found to be impacted by contact sport participation, age, and number of concussions. These biomarkers are implicated in immune response regulation and vascular homeostasis, making their alterations significant in the context of sports-related neuroinflammation and injury.^44,45^ The significantly increased levels of CCL-2 among former contact athletes could suggest a possible association between RHI exposure and heightened neuroinflammatory responses. CCL-2 is a chemokine that is involved in recruiting monocytes and other immune cells to sites of injury or infection.^46^ Prior research has indicated that CCL-2 plays a role in neuroinflammation following TBI, contributing to BBB disruption and leukocyte infiltration into the central nervous system.^47^ The increase in CCL-2 levels among the former contact athletes may reflect a chronic inflammatory state induced by RHI, which could predispose these individuals to long-term neurodegenerative conditions. Additionally, the observed significant association between older age and increased CCL-2 levels aligns with previous findings suggesting that aging is accompanied by heightened systemic inflammation or “inflammaging.”^48^ The interaction between aging and a history of lifetime exposure to sports-related head impacts may exacerbate these inflammatory processes, further increasing vulnerability to neurodegenerative diseases.

Interestingly, while independent-samples *t*-tests indicated no significant group differences in interleukin-2 (IL-2) levels between former contact and noncontact athletes, the inclusion of covariates revealed significantly higher IL-2 levels in former contact athletes. IL-2 is a cytokine crucial for T cell proliferation and immune regulation,^49^ and its elevation could reflect an adaptive immune response to exposure to RHI.^50^ The increased IL-2 levels observed in former contact athletes could indicate an immune-mediated attempt to mitigate neuroinflammation or promote neuronal repair following prolonged exposure to RHI. Notably, athletes with a history of multiple concussions exhibited significantly lower IL-2 levels in our study. This finding suggests that repeated concussions may lead to immune exhaustion or dysregulation, ultimately reducing cytokine production over time. Prior research has linked head trauma to long-term immunosuppression and altered cytokine profiles, which may heighten susceptibility to infections and impair neuroprotection.^51,52^

Chronic systemic inflammation is increasingly recognized as a key contributor to the pathogenesis of dementia, particularly AD.^53,54^ Elevated levels of inflammatory markers such as IL-6, C-reactive protein, and tumor necrosis factor-α have consistently been associated with the acceleration of cognitive decline and increased dementia risk.^55,56^ Although only IL-6 was analyzed in this study and showed only a trend toward being significantly elevated in former contact athletes, its role in chronic neuroinflammatory signaling underscores the potential relevance of even modest elevations in this cohort.^57^ Moreover, the observed increases in other pro-inflammatory cytokines, such as IL-8 and VCAM-1, may similarly reflect sustained immune dysregulation that could contribute to downstream neurodegenerative cascades. These findings highlight the potential long-term neurological risks associated with contact sport participation and support the need for ongoing monitoring of retired contact sport athletes.

It is also important to consider how lifestyle differences between former contact and noncontact athletes may influence the observed outcomes. Noncontact athletes may have sustained higher levels of long-term aerobic fitness, healthier cardiovascular profiles, or have continued to engage in physical activity, all of which have been associated with reduced systemic inflammation and improved neurocognitive outcomes.^58^ Conversely, contact sport athletes may experience higher rates of orthopedic injuries, pain, or reduced physical activity post-retirement, which could contribute to elevated inflammatory burden.^59,60^ These lifestyle factors may partly account for group difference that were observed in the systemic inflammatory markers. Future studies should aim to comprehensively assess lifestyle variables to disentangle their potentially moderating effects on the relationship between RHI and long-term brain health.

### Limitation

The results of this study should be interpreted within the context of several limitations. The sample size was limited with the inclusion of 38 former contact athletes and 18 former noncontact athletes. However, the aim of this study was to explore the utility of brain injury blood biomarkers and inflammatory markers in a chronic neurological state due to years of RHI exposure. Our data will help inform future longitudinal studies with a larger sample size that can inform the clinical significance of increased inflammatory markers we observed in this study. While this study is novel in the sense that we analyzed both male and female amateur-level athletes of their midlife neurological health, more racial and ethnic diversity would increase the generalizability of the results. Additionally, this study exclusively studied the effects of lifetime exposure to sports-related head impacts in athletes, with the exclusion of those with any history of military combat service or training. Separate studies are needed to analyze these same biomarkers and their effects in those with a history of military combat service or training. Moreover, we did not include medical comorbidities that could contribute to chronic systemic inflammation in our data collection. Future studies should include a more comprehensive health history to better account for confounding factors that may influence inflammatory or neurodegenerative biomarkers. Given the limited sample size, especially in females, we did not stratify between females and males in the analysis. A future study to integrate biological factors of sex (e.g., hormones, menstrual cycle phases, menopause) is encouraged to provide mechanistic insights into possible sex differences in neurodegenerative progressions in former athletic populations.

## Conclusions

This study highlights the neurological and systemic inflammatory effects of decades of participation in contact sports. While initial analyses found no significant differences in key brain injury biomarkers (NfL, GFAP, UCH-L1, and tau), we found that older former contact athletes exhibited higher NfL levels, while concussion history correlated with elevated UCH-L1 and tau, reinforcing the long-term impact of recurrent concussions. Systemic inflammatory markers also demonstrated distinct patterns. Contact athletes had significantly higher levels of systemic biomarkers, including IL-8, CCL-2, CCL-3, IL-2, VCAM-1, and S100B, compared with their noncontact counterparts, indicating RHI’s potential effects on systemic inflammatory responses. These findings emphasize the associations between years of RHI exposure, systemic inflammation, and neurodegenerative progression, underscoring the need for long-term biomarker monitoring and research into interventions to mitigate these effects.

### Transparency, rigor, and reproducibility statement

This cross-sectional study included middle-aged amateur-level athletes who participated in at least 10 years of organized contact sports or at least 10 years of organized noncontact sports. We had 38 former contact sport athletes and 18 former noncontact sport athletes who were matched by age and sex. On testing day, a trained phlebotomist obtained 4 mL of venous blood from each participant into an EDTA vacutainer tube. Plasma was separated from blood via centrifuge (1500 *g*, 15 min, 4°C) and stored at −80°C until analysis. Brain injury blood biomarkers, including GFAP, UCH-L1, tau, and NfL, were analyzed by the Human Neurology 4-Plex A assay on a Quanterix SR-X system. Additionally, a Human Magnetic Luminex Assay was designed using the assay service from R&D Systems. The custom Luminex multiplex assay used a 3-fold plasma sample dilution to quantify a panel of inflammatory cytokines (IL-6, IL-8, IL-1α, IL-2), chemokines (CCL-2, CCL-3, CCL-5), neuronal and glial injury markers (BDNF, S100B, NSE-2, α-synuclein), and vascular integrity markers (Ang-1, Ang-2, neuropilin-1, VCAM-1, ICAM-1, thrombomodulin, CD31) in plasma samples. Participants were blinded regarding any study aims and final outcome and will be referred to the publication when it becomes available. Statistical analyses were performed by a team member blinded to the relevant characteristics of the participants. All equipment and software used to perform analyses are widely available from commercial sources. De-identified data from this study and analytic code are available upon reasonable request to the corresponding author (K.K.).

## Abbreviations Used

AD: Alzheimer’s Disease
BDNF: Brain derived neurotrophic factor
CCL-2: C-C motif ligand 2
CCL-3: C-C motif ligand 3
CCL-5: C-C motif ligan 5
CD31: Platelet endothelia cell adhesion molecule-1
CI: Confidence interval
CTE: Chronic traumatic encephalopathy
CTSI: Clinical and Translational Sciences Institute
DAMPs: damage-associated molecular patterns
EDTA: Ethylenediaminetetraacetic acid
GFAP: Glial fibrillary acidic protein
HMGB1: high mobility group box 1
ICAM-1: Intercellular adhesion molecule-1
IL-1: Interleukin-1
IL-1alpha: Interleukin-1alpha
IL-2: Interleukin-2
IL-6: Interleukin-6
IL-8: Interleukin-8
mTBI: Mild traumatic brain injury
NfL: Neurofilament light
NSE-2: Neuron-specific enolase-2
P-tau: Phosphorylated tau
RHI: Repetitive head impacts
TBI: traumatic brain injury
T-tau: Total tau
UCH-L1: Ubiquitin C-terminal hydrolase L1
VCAM-1: vascular cell adhesion molecule-1
