# Supplementary material for: Effects of Lifetime Exposure to Sports-Related Head Impacts on Brain Injury and Inflammatory Blood Biomarkers Among Former Middle-Aged Athletes
Source: Neurotrauma Rep. 2025 Aug 5;6(1):638–50. doi: 10.1177/08977151251362101 (PMC12413257; doi:10.1177/08977151251362101)
Supplement: Supplementary Table S1 [file 08977151251362101_supplementarytables1.docx]

| **Supplemental Table 1**: Changes in non-significant biomarker level relative to non-contact athletes | | |
| --- | --- | --- |
| **Brain-injury blood biomarkers (pg/mL)** | NfL | 0.5 (-1.4, 2.5), 0.575 |
|  | GFAP | -8.3 (-29.5, 12.9), 0.434 |
|  | UCH-L1 | 10.1 (-33.8, 53.9) 0.644 |
|  | Tau | 1.4 (-4.2, 6.9), 0.621 |
| **Systemic inflammatory markers (pg/mL)** | IL-6 | 0.3 (-0.03, 0.57), 0.07 |
|  | Angiopoietin-1 | 2219.7 (-157.0, 4596.3), 0.067 |
|  | Neuropilin-1 | -1596.1 (-23,947.2, 20,755.0) 0.886 |
|  | CCL-5 | 176.8 (-1241.6, 1595.2), 0.803 |
|  | IL-1alpha | -0.52 (-6.3, 5.3), 0.858 |
|  | CD31 | -2653.8 (-5783.0, 475.4), 0.095 |
|  | NSE | -2285.7 (-6440.1, 1868.8), 0.275 |
|  | ICAM-1 | 17,804.0 (-27,942.9, 63,549.9), 0.438 |
|  | Alpha-Synuclein | -6.4 (-59.6, 46.7), 0.809 |
|  | Thrombomodulin | 53.3 (-248.0, 354.7), 0.724 |
| **Note**: values are expressed as difference (95% confidence interval), p-value | | |
