# Supplementary material for: Effects of Lifetime Exposure to Sports-Related Head Impacts on Brain Injury and Inflammatory Blood Biomarkers Among Former Middle-Aged Athletes
Source: Neurotrauma Rep. 2025 Aug 5;6(1):638–50. doi: 10.1177/08977151251362101 (PMC12413257; doi:10.1177/08977151251362101)
Supplement: Supplementary Table S2 [file 08977151251362101_supplementarytables2.docx]

| **Supplemental Table 2**: Average Biomarker Value by Group | | | |
| --- | --- | --- | --- |
|  | | **Contact** | **Non-Contact** |
| **Quanterix Neurology 4-Plex A** | NfL | 7.1 ± 3.8 | 6.2 ± 2.6 |
|  | GFAP | 74.9 ± 33.9 | 82.2 ± 30.4 |
|  | UCH-L1 | 113.5 ± 121.6 | 102.7 ± 151.1 |
|  | Tau | 11.7 ± 12.5 | 8.9 ± 11.6 |
| **Luminex Human Discovery Assay** | IL-6 | 1.2 ± 0.4 | 0.9 ± 0.6 |
|  | Angiopoietin-2 | 544.2 ± 228.5 | 444.5 ± 165.7 |
|  | Neuropilin-1 | 60,402.5 ± 37,721.6 | 62,446.8 ± 37,663.4 |
|  | CCL-5 | 5091.7 ± 2469.7 | 4948.5 ± 2231.0 |
|  | IL-1alpha | 9.2 ± 9.4 | 9.0 ± 10.9 |
|  | CD31 | 6203.6 ± 3672.6 | 8793.2 ± 7833.3 |
|  | NSE | 8063.0 ± 6164.9 | 10,502.7 ± 8178.8 |
|  | ICAM-1 | 144,659 ± 81,439 | 125,618 ± 71,787 |
|  | Alpha-Synuclein | 400.7 ± 104.4 | 404.4 ± 40.6 |
|  | BDNF | 1912 ± 2109.1 | 902.4 ± 1178.2 |
|  | IL-8 | 2.2 ± 1.2 | 1.3 ± 0.8 |
|  | S100B | 130.9 ± 151.8 | 45.0 ± 53.7 |
|  | CCL-3 | 240.2 ± 111.2 | 180.6 ± 87.0 |
|  | VCAM-1 | 270,116 ± 83,708 | 222,988 ± 75,238 |
|  | Angiopoietin-1 | 4068.5 ± 4500.6 | 1889.6 ± 2504.8 |
|  | CCL-2 | 59.4 ± 28.0 | 45.3 ± 20.7 |
|  | IL-2 | 1.2 ± 0.9 | 0.8 ± 0.9 |
|  | Thrombomodulin | 1952.5 ± 536.4 | 1879.2 ± 496.1 |
| **Note**: mean ± SD (pg/mL) | | | |
